# Chronic Granulomatous Disease First Diagnosed in Adulthood Presenting With Spinal Cord Infection

**DOI:** 10.3389/fimmu.2018.01258

**Published:** 2018-06-04

**Authors:** Philipp Schwenkenbecher, Alexandra Neyazi, Frank Donnerstag, Felix C. Ringshausen, Roland Jacobs, Matthias Stoll, Philip Kirschner, Florian Peter Länger, Emil Valizada, Stefan Gingele, Florian Wegner, Kurt-Wolfram Sühs, Martin Stangel, Thomas Skripuletz

**Affiliations:** ^1^Department of Neurology, Hannover Medical School, Hannover, Germany; ^2^Department of Psychiatry, Social Psychiatry and Psychotherapy, Hannover Medical School, Hannover, Germany; ^3^Institute for Neuroradiology, Hannover Medical School, Hannover, Germany; ^4^Department of Respiratory Medicine, Hannover Medical School, German Center for Lung Research (DZL), Hannover, Germany; ^5^Department of Clinical Immunology and Rheumatology, Hannover Medical School, Hannover, Germany; ^6^Institute for Medical Microbiology and Hospital Epidemiology, Hannover Medical School, Hannover, Germany; ^7^Institute of Pathology, Hannover Medical School, Hannover, Germany

**Keywords:** chronic granulomatous disease, spinal cord diseases, immunodeficiency, infectious diseases, cerebrospinal fluid

## Abstract

Chronic granulomatous disease (CGD) is a rare genetic immunodeficiency, which is characterized by recurrent severe bacterial and fungal infections caused by a defect in phagocytic cells due to loss of superoxide production. The disease usually manifests within the first years of life. Early diagnosis allows therapeutic intervention to improve the limited life expectancy. Nevertheless, only half of the patients exceed the age of 25. Here, we present the case of a 41-year old female patient who presented with an extensive spinal cord infection and atypical pneumonia mimicking tuberculosis. The medical history with recurrent granulomatous infections and microbiological findings with multiple unusual opportunistic pathogens was the key to the diagnosis of CGD, which is exceptionally rare first diagnosed in patients in the fifth decade of life. The late diagnosis in this case was likely due to the lack of knowledge of the disease by the treating teams before but not because the patient did not have typical CGD infections along her life. The extensive progressive developing granulomas in our patient with fatal outcome raise the question of early immunosuppressive therapy in addition to anti-infectious treatment. We recommend appropriate CGD diagnostics in adult patients with unclear granulomatous diseases of the nervous system.

## Introduction

Chronic granulomatous disease (CGD) is a genetic immunodeficiency characterized by various recurrent bacterial and fungal infections (1, 2). The cause of the disease is the inability of the neutrophils to dispose certain pathogens due to a defective function of the nicotinamide adenine dinucleotide phosphate (NADPH) oxidase, which produces hydrogen peroxide (1). Hydrogen peroxide is metabolized to reactive oxygen species, which is necessary for the phagocyte respiratory burst that kills the previously ingested pathogens (1, 3–8). However, the majority of pathogens produce hydrogen peroxide independently from the NADPH oxidase (1, 3–8). In some fungi and bacteria such as Aspergillus, Candida, Staphylococcus, and Nocardia, the small amount of hydrogen peroxide is neutralized by a pathogen-inherited catalase (1, 3–8). In the course of an infectious disease, patients with CGD develop granulomas as a result of a dysregulated inflammatory response potentially leading to obstructive lesions (9, 10).

The most common form of CGD is the result of a mutation in an X-chromosome-linked gene and accounts for approximately 80% of all, almost exclusively male, patients (11). Symptoms of these patients usually manifest within the first 2 years of life (4). However, some patients present later in life due to an autosomal-recessive manner (4, 12). Due to its sporadic occurrence in about 1 in 250,000 individuals, the clinical course and outcome have only been partially defined (4). The spectrum of clinical manifestations of CGD comprises infections of the lung, skin and lymph nodes, and the gastrointestinal (GI) tract (13, 14). Pulmonary involvement is most frequent and consists of pneumonia and lung abscesses followed by skin affection, ranging from abscesses in the skin to aseptic granulomas (1, 4). Pathogens isolated in CGD are associated with sites of infections (1). The affection of the central nervous system, e.g., intracranial abscesses presents a rare complication (11, 15–21) and so far only two cases of myelitis had been described in children (15, 22).

Here, we describe the case of a female patient in the fifth decade of life who presented with an extensive spinal cord infection leading to the diagnosis of CGD.

## Case Presentation

**Figure 1:**
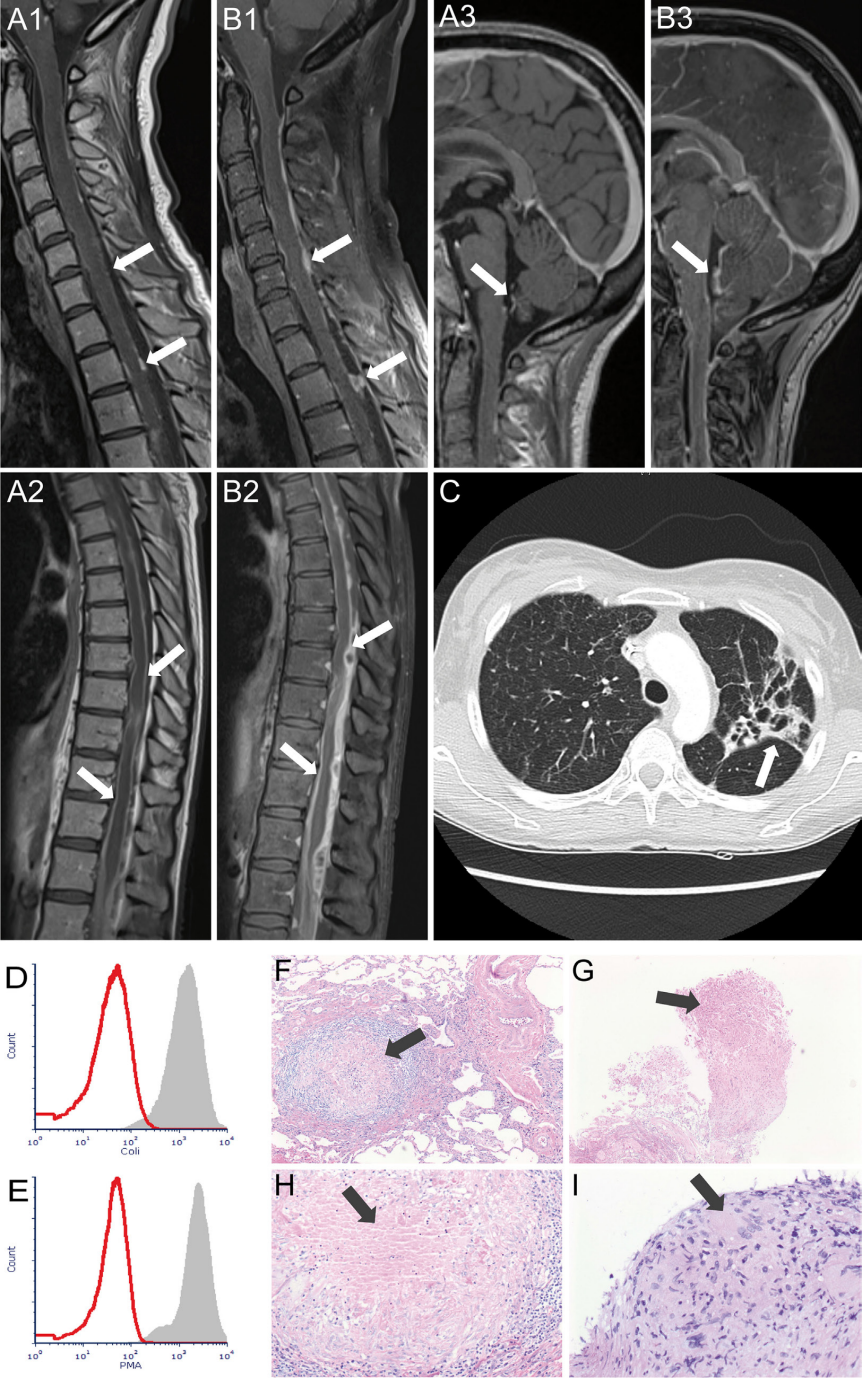
Magnetic resonance imaging demonstrating multiple contrast-enhanced nodular masses along the leptomeninges of cervical **(A1)**, thoracic **(A2**), and brain **(A3)** region at admission and progress in all three regions after 3 weeks **(B1–B3)**. Arrows indicate leptomeningeal enhancement with multiple nodular lesions. Chest computed tomography **(C)** at admission revealed patchy areas of consolidation and cavitation primary in the left upper lung lobe (arrow). **(D,E)** show the respiratory burst capability of neutrophils. 100 µl of heparinized whole blood of the patient (red histograms) and a healthy control (gray histograms) was stimulated with *Escherichia coli* **(D)** and phorbol-myristate acetate **(E)** in a 37°C water bath for 10 min. After adding DHR123, samples were incubated for another 10 min. Finally, the red blood cells were lysed, and the washed samples were subjected to flow cytometry analysis. Histopathological examination (hematoxylin and eosin stain) shows granulomas as indicated by arrows in the lung tissue obtained by surgical biopsy in 2007 **(F,H)** and obtained by bronchoscopy in 2016 **(G,I)**.

A 41-year-old female patient was admitted to a local hospital abroad with a 4-week history of lower respiratory tract infection that had been refractory to antibiotic treatment. Several weeks after initial clinical improvement after intravenous antibiotics against pathogens commonly associated with community acquired pneumonia she developed headache, back pain, and fever. Due to an acute onset of spinal cord symptoms with paraparesis accompanied by bladder and bowel dysfunction she was transferred to our neurological department. Spinal magnetic resonance imaging (MRI) showed multiple contrast enhanced nodular masses along the leptomeninges in the cervical, thoracic, and lumbosacral segments of the spinal cord including the cauda equine and a myelopathy in the thoracic segment (Figures 1A1,A2). A lumbar puncture was performed and revealed an elevated cerebrospinal fluid (CSF) cell count (314 cells/μl), highly increased levels of protein (45,300 mg/l), a severe blood-CSF barrier dysfunction (Q-Albumin 694), and increased lactate concentration (7 mmol/l). Initially, neither bacterial (conventional cultural growth, mycobacterial cultures, *Borrelia burgdorferi*, and *Treponema pallidum* antibodies test), nor viral (herpes simplex virus, varicella zoster virus, cytomegalovirus, Epstein–Barr virus, enteroviruses, measles virus, rubella virus, tick-borne encephalitis, and JC virus), or fungal (cultural growth and antigen test to Aspergillus and *Cryptococcus neoformans*) pathogen could be detected in CSF. Laboratory testing for autoimmune causes such as connective tissue diseases (antinuclear antibodies, anti-DNA antibodies, antiphospholipid antibodies, antineutrophil cytoplasmic antibodies, and rheumatoid factor) was unremarkable. Computed tomography (CT) of the chest showed findings suggestive for tuberculosis (Figure 1C) and tuberculostatic therapy with ethambutol, rifampicin, isoniazid, and pyrazinamide was started. Histopathological examination of lung tissue obtained by bronchoscopic transbronchial biopsy revealed granulomas compatible with tuberculosis (Figures 1G,I).

Another spinal MRI after 2 weeks of treatment exhibited a slight progress in the extension of the nodular masses but a regress of myelopathy. CSF examination revealed a decreased cell count (195 cells/μl) accompanied by an unchanged severe blood–CSF barrier dysfunction (Q-Albumin 742).

Further analyses by using the polymerase chain reaction (PCR) technique detected the atypical *Mycobacterium genavense* in the lung tissue, while this was considered a possible contamination since retesting and cultural growth were negative for this pathogen. Cultural growth of sputum and bronchoalveolar lavage fluid revealed *Serratia marcescens* and *Aspergillus fumigatus*. Thereupon, cotrimoxazole, azithromycin, and voriconazole were added to patient’s treatment. In addition, the patient was medicated with high-dose methylprednisolone to suppress inflammatory bystander reaction, which is considered responsible for developing granulomas (23, 24).

Documents of patient’s medical history could be acquired meanwhile. In 1990s, she was diagnosed with discoid lupus erythematosus due to skin lesions in the face and breast with compatible histological findings. As symptoms regressed, neither further testing was performed nor did she receive any therapy. When she was 28 years old, she suffered from severe pneumonia for 3 months and was treated at an intensive care unit. Histological examination of lung tissue showed granulomas of unspecific origin. Due to positive pANCA testing, vasculitis with lung involvement was considered and the patient was treated with cyclophosphamide followed by azathioprine for several months. However, immunosuppressive therapy was not continued, and follow-up was lost. Four years later, she suffered again from severe life-threatening pneumonia with multiorgan failure. Again granulomas could be found in lung tissue. Nocardia was identified in bronchoalveolar lavage, and as the patient recovered after adaption of antibiotic treatment, further investigations were not performed. The sister of our patient remembered a severe lung infection in the early childhood, which led to hospitalization and was treated with rifampicin.

When we learned about the complex previous medical history, we requested the previous lung tissue samples and compared them with the currently obtained lung tissue samples. Histological assessment revealed similar patterns of granulomas (Figures 1F–H).

Due to recurrent granulomatous infections with the evidence of atypical pathogens, the capacity of patient’s phagocytic leukocytes to produce superoxide or hydrogen peroxide was examined *in vitro* by stimulation with *Escherichia coli* and phorbol-myristate acetate. The analyses revealed a complete loss of intracellular hydrogen peroxide production (Figures 1D,E), which was consistent with the diagnosis of CGD. Genetic testing was performed. The exons 2, 6, 7, and 8 of the neutrophil cytosolic factor 1 (*NCF1*) gene were amplified by using the PCR technique but no PCR product could be obtained, indicating homozygous deletion in the *NCF1* gene which confirmed the diagnosis of autosomal-recessive CGD.

Unfortunately, the patient’s medical condition deteriorated rapidly. Spinal MRI 3 weeks after admission exhibited further progress of the nodular masses (Figures 1B1,B2). She developed an acute disturbance of consciousness and epileptic seizures. CT brain imaging showed brain edema caused by hydrocephalus internus as a consequence of brainstem and cervical spinal cord compression due to nodular masses. External ventricular drainage was applied. Brain edema regressed but MRI of the brain showed signs of brainstem meningitis and ventriculitis (Figure 1B3). PCR analysis of the CSF detected an infection with *Nocardia otitidiscaviarum*. Patient’s medical condition further deteriorated, and CT of the brain showed increasing brain edema despite ventricular drainage. The patient died 30 days after admission to our department.

## Discussion

Here, we report an unusual clinical presentation of a middle-aged female patient with CGD. The case at hand is outstanding in several aspects. (1) The late diagnosis of CGD in patient’s fifth decade of life surviving previous severe infectious diseases is remarkable. (2) Central nervous system manifestation in CGD is rare, but spinal cord manifestation is exceptional. (3) Genetic examination detected a total lack of the *NCF1* sequence, indicating a homozygous deletion, which was described in only a few families to date.

Our patient is one of the few published cases who were diagnosed with CGD at the age older than 40 years. The majority of patients with CGD are diagnosed in early childhood; in only 4% of all cases, the diagnosis is made after the second decade (25–27). This late diagnosis of CGD commonly occurs in patients with the autosomal-recessive heredity, as found in our patient, which manifests itself later in life (3, 14, 27, 28).

The most common autosomal subtype of CGD is caused by mutations in the *NCF1* gene on chromosome 7q11.23, which is responsible for creating the protein NCF1 (also known as p47-phox) (29). In the autosomal-recessive manner, further mutations are found in genes encoding p67phox (*NCF2* on chromosome 1q25) and p22phox (CYBA on chromosome 16q24) (4). These proteins present subunits of proteins that form the enzyme complex NADPH oxidase. P22phox is located in the plasma membrane of the NADPH oxidase, while p47phox and p67phox are cytosolic subunits (4, 30). NADPH oxidase regulates the inflammatory response in phagocytes by producing superoxide, which is mandatory to destroy bacteria and fungi. Measurement of the oxidative burst (superoxide production) of neutrophils in response to stimulation revealed complete inability to produce superoxide in our patient.

Genetic examination confirmed the diagnosis CGD as a total lack of the *NCF1* sequence was found indicating a homozygous deletion, which has been described in only 24 families with CGD (29). In healthy patients, the *NCF1* gene consists of 11 exons (31). The gene locus is accompanied on each side by one pseudo *NCF1* gene (31). Both pseudogenes contain a GT deletion at the start of exon 2 causing a frameshift and premature termination of protein synthesis (29, 31). Unequal cross-over events between *NCF1* and pseudogenes during DNA replication of repair can cause a lack of intact *NCF1* since individuals only have pseudogenes with GT deletion (29, 31). In our patient, the lack of PCR products for the exons 2, 6, 7, and 8 indicates a large deletion, which can be explained by a splice-site mutation in the bordering introns (31).

Chronic granulomatous disease manifests with repeated severe bacterial and fungal infections resulting in the formation of granulomas. The most frequently affected organs are lungs, followed by skin/subcutis, lymph nodes, gastro-intestinal tract, and liver (4, 32). In a large European study, collected data suggested that it is not possible to adequately determine the first symptom or site of disease at presentation (4). This study, however, demonstrated that only 7% of all patients with CGD, who experienced more than one episode of related infections, suffered from brain infections (4). To the best of our knowledge, there are only two other case reports about spinal cord infections in CGD (15, 22). The diagnosis of CGD was challenging and misguiding as initial findings including CT and MR imaging and CSF examination were suggestive for tuberculosis with spinal cord involvement. However, culture growth of bronchoalveolar lavage fluid revealed *A. fumigatus* and *S. marcescens*. Both pathogens were uncommon and could not be identified in three CSF examinations. Nevertheless, antibiotic and antifungal treatments were performed. Together with *N. otitidiscaviarum*, which was identified in the last CSF analysis, our patient was infected with pathogens commonly found in CGD which are *Aspergillus, Candida, Staphylococcus, Serratia*, and *Nocardia* (3, 4). Since *N. otitidiscaviarum* was found in the CSF but not systemic, it remains unclear if this pathogen spread to the CSF or infected the CNS exclusively.

Besides the microbiological findings, the medical history of our patient with recurrent granulomatous pulmonary infections with onset in the early childhood was the major clue for the diagnosis. However, our patient was not diagnosed before with CGD although she had many previous CGD-related complications. The diagnosis was complicated by several issues. Since the diagnosis is usually made in early childhood, pediatricians but not internists or neurologists are familiar with this rare disease. In patients with granulomatous diseases, internists and neurologists expect either an infectious disease such as tuberculosis or an autoimmune disorder. Some autoimmune diseases in which granulomas typically occur such as sarcoidosis can imitate CGD regarding clinical manifestation, imaging, and CSF findings. Furthermore, the prevalence of autoimmune diseases is considered to be even higher in patients with CGD compared to the normal population (33–35). Interestingly, our patient experienced an episode of a discoid lupus erythematosus such as skin lesions in her twenties, which are described in patients with CGD (36, 37). Furthermore, our patient exhibited pANCA antibodies during one previous pneumonia and was thus treated with immunosuppressive therapies including cyclophosphamide. In CGD patients, a dysregulated hyperinflammation is the suspected cause of granulomas, which usually manifests in urogenital, respiratory, or GI tract (23, 24). The manifestation of granulomas in the GI tract can even imitate the inflammatory bowel disease Crohn’s disease (38). Since previous reports suggested the use of steroids in patients with CGD and obstructive symptoms, liver abscesses, and life-threatening hyperinflammatory responses our patient received methylprednisolone (39–43). Although our patient was treated with steroids, the progress of granulomas, which led to hydrocephalus internus causing brain edema, could not be stopped. In our case, granulomas in the central nervous system led to fatal complication.

The diagnosis of CGD in an early stage offers the possibility of long-term, low-dose antibiotics and antifungal prophylactic treatment, as well as interferon γ treatment, which is considered to possess a protective effect against infections (44–46). Hematopoietic stem cell transplantation is currently the only curative treatment for CGD (4).

## Conclusion

Chronic granulomatous disease is a life-threatening genetic immunodeficiency, which has to be diagnosed as early as possible to maintain prophylactic options including long-term antibiotic therapy. Our case shows that CGD is not only a disease of children but can be found even in adult patients. The late diagnosis in our case was likely due to the lack of knowledge of the disease by the treating teams before, but not because the patient did not have typical CDG infections along her life. We thus suggest to perform appropriate CGD diagnostics in adult patients with unclear granulomatous diseases.

## Ethics Statement

The legal representative of the patient gave written informed consent for publication.

## Author Contributions

PS analyzed data and drafted the manuscript. AN, FR, MSto, EV, SG, FW, and K-WS analyzed clinical data. FD analyzed radiological data. RJ analyzed immunological data. PK analyzed microbiological data. FL analyzed pathological data. MSta: analyzed data and contributed in drafting the manuscript. TS conceived the article, analyzed data, and drafted the manuscript.

## Conflict of Interest Statement

The authors declare that the research was conducted in the absence of any commercial or financial relationships that could be construed as a potential conflict of interest.

## Abbreviations

CGD, chronic granulomatous disease; NADPH, nicotinamide adenine dinucleotide phosphate; ROS, reactive oxygen species; GI, gastrointestinal; MRI, magnetic resonance imaging; CSF, cerebrospinal fluid; CT, computed tomography; PCR, polymerase chain reaction; PMA, phorbol-myristate acetate; *E. coli, Escherichia coli*; *NCF1*, neutrophil cytosolic factor 1.
